# Body Surface Ultrastructure as a Main Morphological Criterion for Distinguishing Adult Trematode *Metagonimus suifunensis*

**DOI:** 10.3390/biology13110942

**Published:** 2024-11-18

**Authors:** Polina Shumenko, Yulia Tatonova, Mikhail Shchelkanov

**Affiliations:** 1 Federal Scientific Center of the East Asia Terrestrial Biodiversity, Far Eastern Branch, Russian Academy of Sciences, pr-t 100-letiya Vladivostoka 159a, Vladivostok 690022, Russia; 2 G.P. Somov Research Institute of Epidemiology and Microbiology, Russian Federal Service for Surveillance on Consumer Rights Protection and Human Wellbeing, Selskaya St. 1, Vladivostok 690022, Russia

**Keywords:** SEM, *Metagonimus*, surface ultrastructure, morphology

## Abstract

In the present study, the ultrastructure of the body surface of mature trematodes of *Metagonimus suifunensis* Shumenko et al., 2017 has been studied for the first time. It has been found that the tegumental spines on the surface of their bodies are very different from those in three other species of large trematodes of the genus *Metagonimus*: *M. yokogawai* Katsurada, 1912; *M. takahashii* Suzuki in Takahashi, 1929; and *M. miyatai* Saito et al., 1997. Since other morphological characteristics do not allow us to distinguish between these species, the ultrastructure of tegumental spines can be considered the only reliable morphological feature differentiating this trematode from other representatives of the genus. It has also been suggested that the shape of these spines may correlate with the parasite’s ability to damage the host’s intestinal tissue.

## Simple Summary

The tegument surface of adult worms of *Metagonimus suifunensis* was studied using scanning electron microscopy (SEM) and compared with data from other *Metagonimus* species. It was found that the structure of the tegumental spines of the adult worm *M. suifunensis* differs from that of all three previously studied species. The body surface ultrastructure can be used as a diagnostic morphological criterion differentiating *Metagonimus suifunensis* from other species.

## 1. Introduction

Pathogens of different zoonoses have been observed to be prevalent in the East Asian region, which includes the southern Russian Far East. In East Asia, trematodes belonging to the genus *Metagonimus* are common diseases. Among the most significant food-borne parasites worldwide, three species of this genus—*M. yokogawai*, *M. takahashii*, and *M. miyatai*—are known to cause human metagonimiasis [1]. Additionally, *Metagonimus suifunensis*, an endemic parasite that causes gastrointestinal illnesses, is of immense epidemiological significance in the southern Russian Far East.

In the last few years, based on complex data uniting both morphological characteristics and genetic data, new cryptic species have been described in East Asia, including representatives of the genus *Metagonimus* [2,3,4]. Some of these species have morphological differences, while others are practically indistinguishable morphologically and are not even differentiated on the basis of nuclear markers. The same applies to *Metagonimus suifunensis* (Heterophyidae), described as an endemic species to the south of the Russian Far East [2]. In previous studies, this species was identified as *Metagonimus yokogawai* on the basis of morphometric data [5,6,7]. The segregation of this trematode into a separate species was mainly supported by molecular markers. Shumenko and co-authors [2] proved the genetic differences between *M. suifunensis* from Russia and other representatives of the genus *Metagonimus* collected from China, Japan, and South Korea. Subsequently, Tatonova and co-authors [8] obtained nucleotide sequences of the mitochondrial *cox1* gene for *M. suifunensis*, which were compared with those of other representatives of the genus. Further analysis showed that the nucleotide sequences of this marker in *M. suifunensis* differed from the sequences of the closely related species by 13–16%. The data obtained have confirmed the status of *M. suifunensis* as a separate taxonomic unit. Nevertheless, for species identification, besides genetic data, it is also important to consider morphological features. As noted above, the morphological and morphometric characteristics of adult *M. suifunensis* are similar to those of three other representatives of the genus, *M. yokogawai*, *M. takahashii*, and *M. miyatai* [2].

In this regard, the aim of this study was to find additional morphological criteria to differentiate closely related species. The SEM studies of the body surface have been performed for quite a long time for representatives of the superfamily Opistorchioidea [9,10,11], and such data may be especially essential for differentiating closely related trematode species. For this reason, we examined the tegument surface in adult *M. suifunensis* by the SEM method and compared it with the surface ultrastructure of *M. yokogawai*, *M. takahashii*, and *M. miyatai*. We determined whether *M. suifunensis* differs from phylogenetically related species via the structure and location of its tegumental spines.

## 2. Materials and Methods

Samples from the experiment described in [3] were used in the present study. Fish naturally infected by metacercariae from the Odyr River, Khabarovsk Region, Russia, were fed to a laboratory rat. On day 11 post-feeding, the rat was anesthetized with chloroform and subjected to autopsy. The keeping and euthanasia of all the animals were carried out in accordance with the decision of the Committee on the Ethics of Animal Experiments, Federal Scientific Center of East Asia Terrestrial Biodiversity (FSCEATB), Far Eastern Branch, Russian Academy of Sciences (FEB RAS) (Permit No. 1 of 25 April 2022). Trematodes isolated from the rat’s intestine and identified as *Metagonimus suifunensis* under a microscope were rinsed in a 0.9% physiological solution of sodium chloride. For preparing SEM micrographs, the trematodes were fixed in 96% ethanol and critical point dried using a Quorum K850 device (Quorum Technologies, Ltd., Lewes, UK). Then, the specimens were sputter-coated with carbon in a layer approximately 4–6 nm thick by a Quorum Q150TES sputter coater (Quorum Technologies, Ltd., Lewes, UK). Electronic micrographs of the trematodes were taken through a MERLIN field emission SEM microscope (Carl Zeiss, Jena, Germany) at the Instrumental Centre for Biotechnology and Gene Engineering, Federal Scientific Center of the East Asia Terrestrial Biodiversity, Far Eastern Branch, Russian Academy of Sciences (Vladivostok, Russia). Micrographs of a total of seven specimens were studied. Then, we compared the shapes of spines from different body parts of the adult *M. suifunensis* with photographs of *M. yokogawai*, *M. takahashii*, and *M. miyatai* available in published data [10,12,13]. The species of these parasites were identified on the basis of the *cox1* mtDNA gene.

## 3. Results

**Figure 1:**
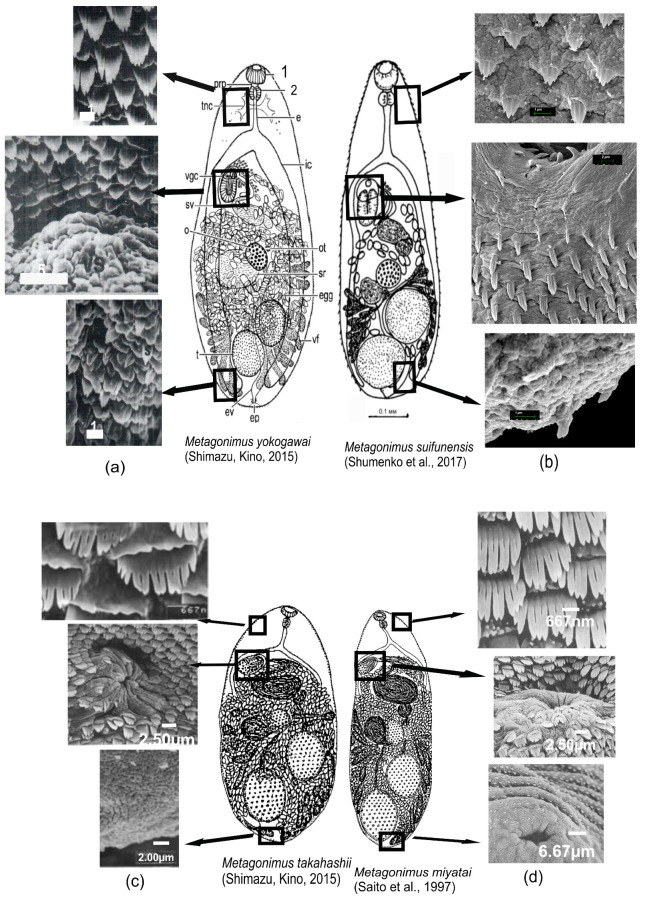
Tegumental spines from the anterior, middle, and posterior ventral parts of (**a**) *M. yokogawai* (Lee et al., 1984 [10]) adult—Shimazu, Kino, 2015; (**b**) *M. suifunensis* (this study), adult—Shumenko et al., 2017; (**c**) *M. takahashii* (Chai et al., 2000 [13]), adult—Shimazu, Kino, 2015 and (**d**) *M. miyatai* (Chai et al., 1998 [12]), adult—Saito et al., 1997.

The body is oval-shaped and elongated, with a tapered anterior end. The oral sucker is transversely oval-shaped and subterminal. The ventral sucker is located to the right of the median line of the body. The body surface is covered with tegumental spines. The arrangement of the spines and their structure varies between different parts of the body. At the anterior end of the body, both on the dorsal and ventral sides, spines are densely distributed, spatulate, wide, and digitated on the upper margin, with 3–5 points adjoining tightly to each other. The ventral sucker is surrounded by numerous digitated spines with 3–5 points. In contrast to the spines located at the anterior end, these are beak-shaped and have a smaller base width. Towards the posterior end of the body, the size of the spines and the number of points in them gradually decrease. At the posterior end, spines are located sparsely and are narrower than those in the anterior and middle parts of the body, almost undivided, but with 2–3 tips at the anterior end of each spine; beak-shaped spines are absent (Figure 1).

## 4. Discussion

Studies performed using electronic microscopy provide data that allow for more accurate identification of trematode species. Regarding the superfamily Opisthorchioidea, to which *M. suifunensis* belongs, data on the ultrastructure of the body surface, in particular, the fine structure of tegumental spines, are available for *Stictodora fuscatum* (Onji and Nishio, 1916) Yamaguti, 1958 [14], *Pygidiopsis summa* Onji and Nishio, 1916 [15], *Heterophyopsis continua* Onji and Nishio, 1916 [16], *Heterophyes nocens* Onji and Nishio, 1916 [12], *Opisthorchis felineus* (Rivolta, 1884) Blanchard, 1895 [17], and other representatives of the superfamily Opisthorchioidea [11,14,18,19]. For the majority of the species of trematodes in the superfamily Opisthorchioidea, the authors proposed considering the quantity and distribution of tegumental spines as diagnostic features of these trematodes. The number of points on the tegumental spines can be used in the taxonomic differentiation of, for example, species within the family [20]. For example, *Heterophyes nocens*, representative of the family Heterophyidae, to which the genus *Metagonimus* belongs, has 12–17 pointed spines on the ventral surface and 15–20 pointed spines on the dorsal surface [21], while *H. continua* is characterized by spines on the ventral and dorsal surfaces with 15–17 pointed tips [16]. Thus, there is a difference in the structure of tegumental spines for different representatives of the genus *Heterophyes*.

On the other hand, for many Opisthorchioidea trematodes, a change in the number of points on the tegumental spines was noted in trematodes of different ages [12]. Therefore, in the present study, flukes of the same age were compared. It should also be taken into account that the spines are located more densely at the anterior end of the body; towards the posterior end, they become more sparse, and their size and digitation decrease [14,18,22,23]. In this regard, the shape, density of arrangement, and number of points on the spines can only be compared in the same parts of the trematode body.

All Asian representatives of the genus *Metagonimus* can be divided into two groups with different body sizes: large (with a body length greater than 0.65 mm) and small (smaller than 0.65 mm), which were confirmed by molecular data [2]. Both according to morphological characteristics and according to molecular data based on the nuclear 28S rRNA gene, *M. suifunensis* is closest to the Asian species *M. yokogawai*, *M. takahashii*, and *M. miyatai* [2,23]*. Metagonimus suifunensis*, as well as *M. yokogawai*, *M. takahashii*, and *M. miyatai*, for which descriptions of the body surface ultrastructure are available (Figure 1), belongs to the group of “large” trematodes. A comparison of genus *Metagonimus* species, being similar in morphological and morphometric characteristics, revealed differences in details of the fine structure of tegumental spines between different parts of the body surface. The oral sucker spines in *M. suifunensis* are more pointed than those in *M. yokogawai* and *M. takahashii*. The spines at the ventral surface of the anterior end of the body in *M. suifunensis* are narrower and more convex than those of *M. yokogawai*, consisting of 3–5 points, with the boundaries between them being featureless, in contrast to the spines of *M. yokogawai* that consist of 7–9 well-expressed points [10]. The spines on the same part of the body in *M. miyatai* and *M. takahashii* are comb-shaped, consisting of 9–12 and 9–11 points, respectively, with their free ends not adjoining tightly. The spines at the ventrogenital complex in *M. suifunensis* are narrower and more pointed than those in the above-listed species; they consist of 3–4 points tightly adjoining each other. The tegumental spines near the ventral sucker in *M. yokogawai* also consist of tightly adjoining points, but the number of such points reaches 5–6; the spines are rounded [10]. The spines in *M. miyatai* and *M. takahashii* in the same part of the body are wider and more rounded than those in *M. suifunensis* and *M. yokogawai*. On the ventral surface at the posterior end of the body, spines in the trematodes from the Russian Far East are narrow and sparsely distributed, almost undivided, with 2–3 points on the margin of each spine. In the same area of the body, the spines in *M. yokogawai* have 2–5 points, while the spines in *M. takahashii* and *M. miyatai* have more points, 5–7 and 5–6, respectively [13]. With regard to *Metagonimus* species, all Asian species had spines that were generally similar in arrangement but varied in shape and their number of points [10,11,12,13], and *M. suifunensis* differs from *M. yokogawai*, *M. takahashii*, and *M. miyatai*. In view of the above findings, among morphological characters, the body surface ultrastructure is currently the only diagnostic morphological feature that reliably differentiates *M. suifunensis* from “large” representatives of the Asian *Metagonimus*, in addition to the previously described molecular markers [2,3].

Regarding the recently obtained data for the species *Metagonimus romanicus* (Ciurea, 1915) Ransom, 1920, and *M. ciureanus* (Witenberg, 1929), described in Europe, it was shown that these representatives of the genus differ significantly from all Asian species both in morphological (including the structure of tegumental spines) and molecular data [23]. The structure of the tegument of both parasites is similar to that of other trematodes in the arrangement of tegumental spines, which are more numerous and divided into outgrowths in the anterior part of the body and become sparse with fewer outgrowths towards the posterior end. However, the number of points differs: the tegumental spines of *M. romanicus* are comb-shaped, with 6–9 points anteriorly, 4–8 points in the middle part, and 2–4 points in the posterior part; the spines of *M. ciureanus* are also comb-shaped, with 5–10 points anteriorly, 4–9 points in the middle part, and with 3–5 points in the posterior part [23]. Moreover, despite these species having “large” body sizes (>0.65 mm), they occupy the external position of all the Asian *Metagonimus* species. *Metagonimus romanicus* differs from the Asian species in the 28S and *cox1* gene data by 1.2–3.8% and 15.9–20.2%, respectively. For *M. ciureanus*, only the nucleotide sequences of the 18S rRNA gene and complete ITS region are available, according to which the species form a branch that is separate from other representatives of the genus [23].

It is likely that the differences in the ultrastructure of spines on the body surface of different representatives of the genus can be considered not only as diagnostic features but also as a factor causing different degrees of damage to the intestine of the definitive host. For example, Ivanskikh [17], in his study on *Opisthorchis felineus*, indicates that, along with the commonly considered features, variation in the arrangement of spines is observed between individuals of the same species, which has an effect on the degree of damage to the epithelium of bile ducts where trematodes are localized. Sohn et al. [18] also noted that more digitated spines in the anterior part of the body are important for the anchorage of *A. felis* in the host intestine. This may also be indicated by the fact that the abdominal and oral suckers are small relative to the body [18]. Antonelli and co-authors [20] also mentioned that the squamosal tegument of *Afallus tubarium* is critical during the process of the attachment of the parasite to the host and maintaining its position in the host intestine. Tegumental spines are likely involved in the abrasion of the host tissues for food and, to a lesser extent, for keeping the animals in place after settling into their extreme habitat [20]. Since the individuals of *M. suifunensis* in the present study had narrower spines, it can be assumed that they caused less damage to the mucosa than the wider spines of *M. yokogawai*, *M. takahashii*, and *M. miyatai*. The latter two, according to this hypothesis, should damage the host tissue to the greatest extent.

## 5. Conclusions

Thus, despite the great morphological similarity of *M. suifunensis* with other representatives of the genus circulating in East Asia, scanning microscopy revealed that the ultrastructure of the spines on the surface of the body in the mature stage is the main differentiating criterion separating the trematodes from those in Russia. In this regard, the data obtained can be used not only for diagnostic purposes but also to further test and compare the effects that *Metagonimus* trematodes have on their definitive host.

## Data Availability

The data presented in this study are available on request from the corresponding author.
